# Supplementary material for: Predictive features of ligand‐specific signaling through the estrogen receptor
Source: Mol Syst Biol. 2016 Apr 23;12(4):864. doi: 10.15252/msb.20156701 (PMC4848761; doi:10.15252/msb.20156701)
Supplement: Supplementary file 1 — Appendix [file MSB-12-864-s001.pdf]

APPENDIX

Predictive Features of Ligand-specific Signaling through the Estrogen Receptor

Jerome C. Nwachukwu[1a], Sathish Srinivasan[1a], Yangfan Zheng[2], Song Wang[2], Jian Min[2], Chune Dong[2], Zongquan Liao[2], Jason Nowak[1], Nicholas J. Wright[1], Rene Houtman[3], Kathryn E. Carlson[4], Jatinder S. Josan[5], Olivier Elemento[6], John A. Katzenellenbogen[4]*, Hai-Bing Zhou[2]*, Kendall W. Nettles[1]*

[1]Department of Cancer Biology, The Scripps Research Institute, 130 Scripps Way, Jupiter, Florida, 33458 USA

[2]Key Laboratory of Combinatorial Biosynthesis and Drug Discovery (Wuhan University), Ministry of Education, Wuhan University School of Pharmaceutical Sciences, Wuhan, 430071, China

[3]PamGene International, Wolvenhoek 10, 5211HH Den Bosch, The Netherlands

[4]Department of Chemistry, University of Illinois, 600 South Mathews Avenue, Urbana, Illinois 61801 USA

[5]Department of Chemistry, Virginia Tech, Blacksburg, VA 24061-0001 USA

[6]Department of Physiology and Biophysics, Institute for Computational Biomedicine, Weill Cornell Medical College, 1305 York Avenue, New York, NY, 10021 USA

[a] Contributed equally

*Correspondence to: Kendall Nettles, knettles@scripps.edu

*Chemistry correspondence to: John Katzenellenbogen, jkatzene@illinois.edu, or Hai-Bing Zhou, zhouhb@whu.edu.cn

Table of Contents

Supplementary Methods

Supplementary Figures: Figure S1

Supplementary Methods

Cell Culture

HepG2, MCF-7, HEK293-T and Ishikawa cells obtained from American Type Culture Collection (ATCC) were cultured in Dulbecco's minimum essential medium (DMEM) (Cellgro by Mediatech, Inc. Manassas, VA) supplemented with 10% fetal bovine serum (FBS) (Hyclone by Thermo Scientific, South Logan, UT), and 1% non-essential amino acids (Cellgro), Penicillin-Streptomycin-Neomycin antibiotic mixture and Glutamax (Gibco by Invitrogen Corp. Carlsbad, CA), were maintained at 37°C and 5% CO_2.

Luciferase (Luc) Reporter Assays

Cells were plated in 10 cm dish, and transfected the next day as follows: HepG2 cells – 10 μg of 3X ERE-luciferase reporter and 1.6 μg of ERα-FL, ERα-ΔAB expression vector, ERα-ΔF, or ERβ; Ishikawa cells – 10 μg 3X ERE-luciferase using FugeneHD reagent (Roche Applied Sciences, Indianapolis, IN). The next day cells were trypsinized, plated onto 384 well plates (Greiner Bio-one, Manro, NC) in 25 μl phenol red-free growth media supplemented with 10% charcoal-dextran sulfate-stripped FBS in triplicates and incubated overnight. After 24 hr cells were stimulated with compounds dispensed using 100 nl pintool Biomeck NXP workstation at 10 μM final concentration (Beckman Coulter Inc.). Luciferase activity was measured after 24 hr using BriteLite reagent (PerkingElmer Inc., Shelton, CT) according to manufacturer's protocol.

Mammalian 2-Hybrid (M2H) Assays

To each well of a 384-well plate 15 μl OptiMEM containing 10 ng of ERα-VP16, 20 ng of either GAL4-NCOA2 or GAL4-NCOA3, 10 ng of 5X GAL4-UAS-luciferase, 30 ng of pSPORT6 and 0.225 μl of Mirus LT1 transfection reagent was plated and incubated for 20 minutes. To this 15 μl of HEK293-T cells (10,000 cells per well) in phenol red free growth media with 10% charcoal-dextran sulfate-stripped FBS was added and incubated overnight. The next day cells were stimulated with compounds dispensed using 100 nl pintool Biomeck NXP workstation at 10 μM final concentration (Beckman Coulter Inc.). Luciferase activity was measured after 24 hr using BriteLite reagent (PerkingElmer Inc., Shelton, CT) according to manufacturer's protocol.

Proliferation Assays

MCF-7 cells were plated on 384-well plates in phenol red free DMEM with 10% FBS and stimulated with compounds after 7 hr using 100 nl pintool Biomeck NXP workstation at 10 μM final concentration (Beckman Coulter Inc.). Treatment was repeated again on the fourth day. Seven days after the initial treatment, 25 µl of CellTitre-Glo reagent (Promega) was added and luminescence was measured. Data was normalized using cell numbers estimated from a cell growth curve generated from cells plated on the seventh day.

Quantitative RT-PCR

MCF-7 cells were plated in 384-well plates in 25 µl phenol red-free growth media supplemented with 5% charcoal-dextran sulfate-stripped FBS at a density of 2500 cells/well in triplicates. Three days later cells were stimulated with compounds for 24 hr. Total RNA was extracted with RNAgem Tissue Plus RNA extraction kit (Zygem, New Zealand). Cells were washed once with PBS, lysed with 7.5 µl RNAgem extraction reagent at 75°C for 5 minutes. Genomic DNA was removed with DNase I treatment for 5 minutes at 37°C and stopped by incubating at 75°C. 5ul of this mixture was used for cDNA synthesis in a total volume of 10 µl using High-Capacity cDNA Reverse Transcription Kit (Life Technologies, Grand Island, NY). The cDNA was diluted 3 times and 4 µl of diluted cDNA was used in qPCR reaction using TaqMan Gene Expression Master Mix (Life Technologies, Grand Island, NY) in a total reaction volume of 10 µl. The qPCR reaction was carried out in a duplex format using pre-designed real-time qPCR assays (Life Technologies, Grand Island, NY) with GAPDH primers with a VIC probe as an endogenous control and *GREB1* primers with FAM probe. The reactions were run on ABI 7900 HT Real-Time PCR system and the fold change relative to vehicle treated samples were calculated using ΔΔCt method.

Chromatin Immuno-precipitation (ChIP) Assay

MCF-7 cells cultured for 3 days in phenol red-free DMEM supplemented with 10% steroid-free FBS, were stimulated with 100 nM E2 or 5 μM WAY-C analogs. ChIP assay was then performed as previously described (Nwachukwu et al., 2014). Essentially, the cells were fixed, rinsed in cold 1X PBS, lysed on ice for 1 hr, sonicated, and subject to immunoprecipitation (IP) overnight at 4°C using NCOA3 antibody (anti-SRC3 M-397, Santa Cruz Biotechnology, Inc.) and Dynabeads protein G (Invitrogen). The precipitate was washed sequentially in low salt, high

salt and LiCl buffers, rinsed twice in 1X TE buffer. The input and IP samples were incubated with 1X TE buffer supplemented with proteinase K (Qiagen, Inc., Valencia, CA) at 65°C for 2.5 hrs to reverse the crosslink, and cleaned up using the QIAquick PCR purification kit (Qiagen). DNA fragments were analyzed by Taqman gene expression assay (Life technologies) using PCR primers and hybridization probes for the *GREB1* promoter (ERE 1): Forward: 5′-GTGGCAACTGGGTCATTCTGA-3′; Reverse: 5′-CG ACCCACA GAAATGAAAAGG-3′; and FAM-probe: 5′-CGCAGCAGACAATGATGAAT-3′.

MARCoNI coregulator interaction profiling

Microarray assay for real-time nuclear receptor coregulator interaction (MARCoNI) was performed as previously described(Aarts et al., 2013). In short, a PamChip peptide micro array with 154 unique coregulator-derived NR interaction motifs (#88101, PamGene International) was incubated with his-tagged ERα LBD in the presence of 10μM compound or solvent only (2% DMSO, apo). Receptor binding to each peptide on the array was detected using fluorescently labeled his-antibody, recorded by CCD and quantified. Per compound, three technical replicates (arrays) were analyzed to calculate the log-fold change (modulation index, MI) of each receptor-peptide interaction versus apo. Significance of this modulation was assessed by Student's t-Test.

Synthetic protocols

Unless otherwise noted, reagents and materials were obtained from commercial suppliers and were used without further purification. Tetrahydrofuran and toluene were dried over Na and distilled prior to use. Dichloromethane was dried over CaH_2 and distilled prior to use. ^{1}H NMR and ^{13}C NMR spectra were obtain on Bruker Biospin AV400 (400 MHz) instrument. The chemical shifts are reported in ppm and are referenced to either tetramethylsilane or the solvent. High-resolution mass spectra data were obtained on IonSpec 4.7 Tesla FTMS. Glassware was oven-dried, assembled while hot, and cooled under an inert atmosphere. Unless otherwise noted, all reactions were conducted in an inert atmosphere. Reaction progress was monitored using analytical thin-layer chromatography (TLC). Visualization was achieved by UV light (254 nm). Flash chromatography was performed with silica gel (0.040-0.063 mm) packing. Elemental analyses were performed on Vario EL III. Melting points were obtained on X-4 melting point apparatus and are uncorrected.

Compounds **7a**-**r** with a BSC appended on the phenol substituents at the C-5 or C-6 positions of the bicyclic core unit were conveniently prepared in two steps (**Scheme 1**). In the first step, a Diels-Alder reaction of 3,4-bis-(4-hydroxyphenyl)furan **8a** with a variety of dienophiles **9** under neat conditions gave the OBHS derivatives **6a-p** in good yield, following an improvement on a known procedure (Liu et al., 2008; Zhou et al., 2005). In the second step, the resulting Diels-Alder adducts **6** were submitted to monoalkylation with K_2CO_3 in acetone using 1.2 equivalents of 2-(N-piperidinoethyl) chloride hydrochloride (for BSC) or in a few cases 2-dimethylaminoethyl chloride hydrochloride (for BSC')(Amari et al., 2004; Shagufta et al., 2006). This step produces a mixture of monoalkylated regioisomers (*ca* 1:1) **7a**-**r** (yield 25-35%) and is accompanied by about 15-25% of dialkylated byproducts (**Scheme 1**). The monoalkylated products **7a**-**r** could be readily isolated and were characterized spectroscopically as a mixture of racemic regioisomers. As has been described previously, the isolated products are the *exo* diastereomers.(Zhou et al., 2005) Importantly, the crystallization of ERα containing OBHS selected for binding of one of the four isomers, which was also consistent in structures of six other OBHS analogs bound to ERα (**Fig. EV5A**).

Compounds **7s** and **7t** were prepared using the Diels-Alder reaction of 3,4-bis-(4-hydroxyphenyl) furan **8a** with dienophiles **9s** and **9t**, which were prepared from ethenesulfonic acid 4-hydroxyphenyl ester **9r** by installation of a basic side chain (**scheme 2A**). Three analogs, **7u**-**w**, which retain a phenolic hydroxy group on the sulfonate phenyl ring while the 'A ring mimic' hydroxyl group was retained (**7u**), removed (**7v**), or replaced by a methoxyl group (**7w**), were prepared via an alternative approach. Diarylfurans **8a-c** with BSCs were prepared first using our previous procedure,(Zheng et al., 2012) and then reacted with **9r** via a Diels-Alder reaction to afford the target compounds **7u-w** in moderate yields (**Scheme 2B**).

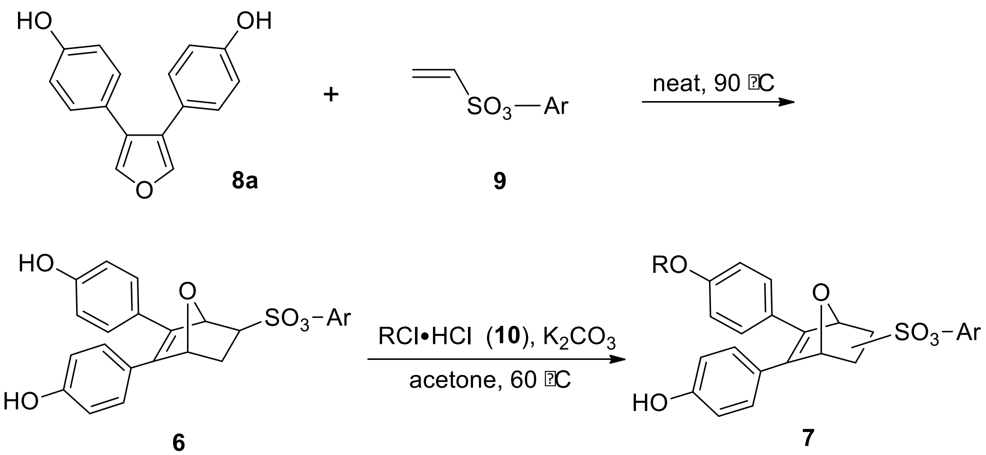

cmpd	Ar
**6a**	Ph
**6b**	2-F-C_6H_4
**6c**	2-Cl-C_6H_4
**6d**	2-Br-C_6H_4
**6e**	3-F-C_6H_4
**6f**	3-Cl-C_6H_4
**6g**	3-Br-C_6H_4
**6h**	4-F-C_6H_4
**6i**	4-Cl-C_6H_4
**6j**	4-Br-C_6H_4
**6k**	2-CH_3-C_6H_4
**6l**	2-Et-C_6H_4
**6m**	2-CH_3O-C_6H_4
**6n**	1-naphthyl
**6o**	2-naphthyl
**6p**	4-CH_3O-C_6H_4

cmpd	Ar	R	conv.	yield
**7a**	Ph	BSC	90%	42%
**7b**	2-F-C_6H_4	BSC	61%	28%
**7c**	2-Cl-C_6H_4	BSC	77%	31%
**7d**	2-Br-C_6H_4	BSC	71%	33%
**7e**	3-F-C_6H_4	BSC	62%	27%
**7f**	3-Cl-C_6H_4	BSC	72%	36%
**7g**	3-Br-C_6H_4	BSC	65%	32%
**7h**	4-F-C_6H_4	BSC	62%	30%
**7i**	4-Cl-C_6H_4	BSC	69%	33%
**7j**	4-Br-C_6H_4	BSC	81%	39%
**7k**	2-CH_3-C_6H_4	BSC	74%	35%
**7l**	2-Et-C_6H_4	BSC	84%	39%
**7m**	2-CH_3O-C_6H_4	BSC	80%	34%
**7n**	1-naphthyl	BSC	87%	40%
**7o**	2-naphthyl	BSC	78%	32%
**7p**	4-CH_3O-C_6H_4	BSC	76%	37%
**7q**	Ph	BSC'	62%	25%
**7r**	4-CH_3O-C_6H_4	BSC'	68%	28%

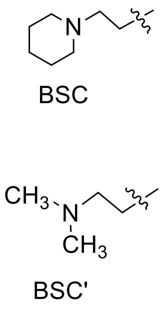

Scheme 1. Synthesis of BSC-containing OBHS Derivatives **7a-r**

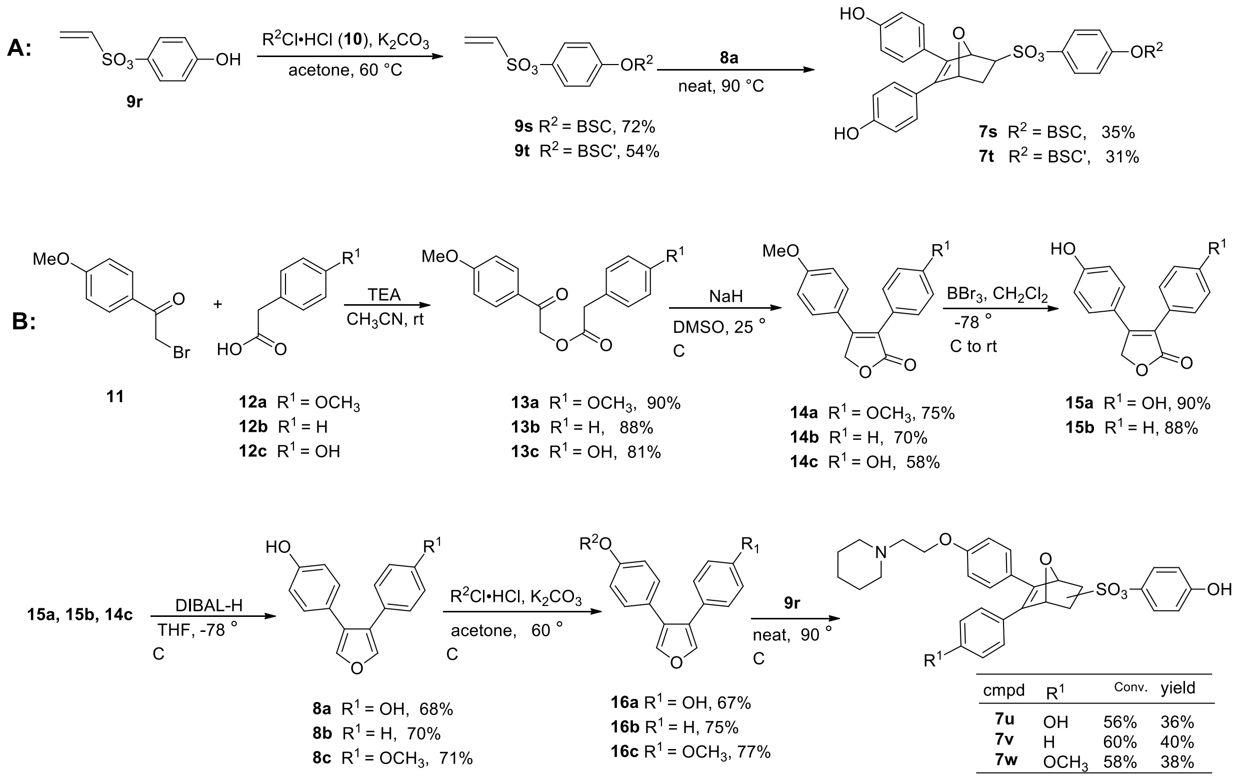

Scheme 2. Synthesis of BSC-containing OBHS Derivatives **7s-w**

Synthesis of the Diaryl-Thiophene Dioxide Series (3,4-DTPD)

Under argon atmosphere, *m*-CPBA (3.0 equivalents) was dissolved in CH_2Cl_2 and added drop-wise to a solution of corresponding thiophenes (1 equiv) in dry CH_2Cl_2 at -20 °C. Then the reaction was allowed to warm slowly to r.t. and stirred overnight. The resulting yellow suspension was diluted with dichloromethane and washed with saturated aqueous sodium bicarbonate, water and brine. The solution was dried over sodium sulfate and concentrated under vacuum. The residue was purified by flash column chromatography.

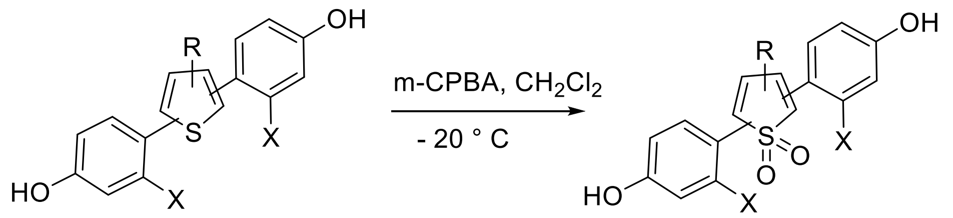

X = F, Cl, Me; R = H, Me

3, 4-Bis(4-hydroxyphenyl)thiophene 1,1-dioxide (MJ-SO1, KWN-2012-01)

^{1}H NMR (400 MHz, acetone-d_6) δ 7.04 (d, J = 8.4 Hz, 4H), 6.83 (d, J = 4.6 Hz, 4H), 6.80 (s, 2H). ^{13}C NMR (101 MHz, acetone-d_6) δ 171.04, 159.91, 145.47, 131.28, 127.36, 123.85, 116.02, 115.94, 60.60.

HR MS (ESI+) (M+1) for $C_{16}H_{13}O_4S$ found 301.0534, calculated 301.0535.

3, 4-Bis(4-hydroxy-2-methylphenyl)thiophene 1,1-dioxide (MJ-SO2, KWN-2012-02)

^{1}H NMR (400 MHz, acetone-d_6) δ 6.92 – 6.75 (m, 4H), 6.65 (d, J = 2.2 Hz, 2H), 6.55 (m, 2H), 2.10 (s, 6H). ^{13}C NMR (101 MHz, acetone-d_6) δ 159.04, 146.03, 138.61, 131.51, 128.94, 123.79, 117.99, 113.31, 20.40. HR MS (ESI+) (M+1) for $C_{18}H_{17}O_4S$ found 329.0846, calculated 329.0848.

3, 4-Bis(2-fluoro-4-hydroxyphenyl)thiophene 1,1-dioxide (MJ-SO3, KWN-2012-03)

^{1}H NMR (400 MHz, acetone-d_6) δ 6.91 (t, J = 8.6 Hz, 1H), 6.86 (s, 1H), 6.53 (dd, J = 8.5, 2.3 Hz, 1H), 6.44 (dd, J = 12.0, 2.3 Hz, 1H). ^{13}C NMR (101 MHz, acetone-d_6) δ 159.56, 158.29, 147.96, 129.44, 116.03, 114.17, 104.20, 103.13. HR MS (ESI+) (M+1) for $C_{16}H_{11}O_4F_2S$ found 337.0348, calculated 337.0346.

3, 4-Bis(2-chloro-4-hydroxyphenyl)thiophene 1,1-dioxide (MJ-SO4, KWN-2012-04)

^{1}H NMR (400 MHz, acetone-d_6) δ 7.04 – 6.90 (m, 2H), 6.82 (d, J = 14.0 Hz, 2H), 6.69 (t, J = 9.1 Hz, 2H), 6.66 (s, 2H). ^{13}C NMR (101 MHz, acetone-d_6) δ 160.10, 143.68, 134.05, 132.59, 130.09, 122.79, 117.25, 114.87. HR MS (ESI+) (M+1) for $C_{16}H_{11}O_4SCl_2$ found 368.9760, calculated 368.9755.

2, 3-Bis(2-chloro-4-hydroxyphenyl)thiophene 1,1-dioxide (MJ-SO9, KWN-2012-09)

^{1}H NMR (400 MHz, acetone-d_6) δ 7.56 (d, J = 8.5 Hz, 1H), 7.33 (d, J = 7.0 Hz, 1H), 7.14 (d, J = 7.0 Hz, 1H), 6.98 (m, 2H), 6.95 – 6.84 (m, 2H), 6.70 (dd, J = 8.5, 2.3 Hz, 1H). ^{13}C NMR (101 MHz, acetone-d_6) δ 160.68, 160.59, 137.04, 135.87, 135.80, 134.60, 134.25,133.62, 132.53, 129.69, 123.89, 117.84, 117.80, 117.33, 115.60, 115.38. HR MS (ESI+) (M+1) for $C_{16}H_{11}O_4SCl_2$ found 368.9756, calculated 368.9755.

Supplementary Figures

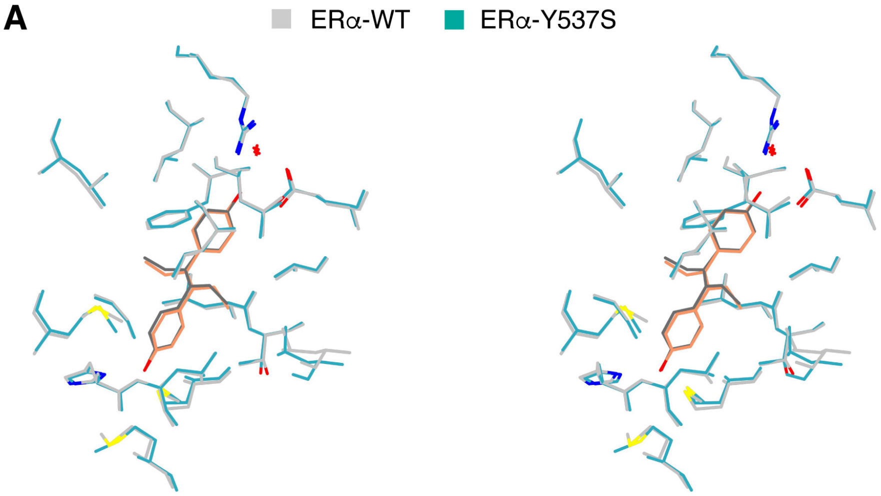

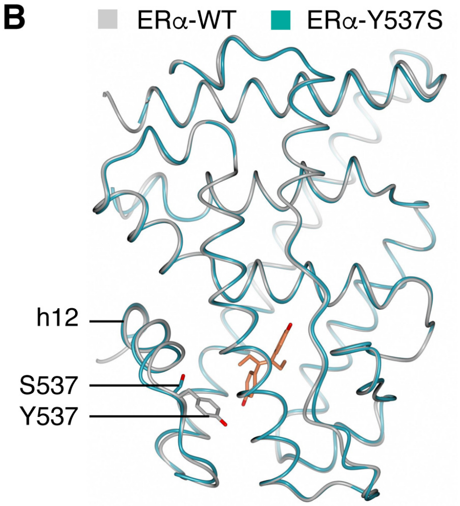

C ERα-Y537S LBD structures

<u>New structures</u>	<u>65</u>
A-CD	4
Cyclofenil	20
2,5-DTP	2
3,4-DTPD	2
Furan	1
Imine	6
Imidazopyridine	2
OBHS	6
*OBHS-N	8
S-OBHS-2	2
S-OBHS-3	1
Triaryl-ethylene	10
DES	1
<u>Previously published</u>	<u>11</u>
A-CD	1
2,5-DTP	1
OBHS	1
WAY-C	4
WAY-D	4
Total	76

Figure S1. Crystal structures of ligand-bound ERα-Y537S LBD complexes

A Structures of DES-bound ERα-WT and -Y537S LBDs were superposed (PDB 3ERD and 4ZN7). A stereo view of the ligand-binding pocket is shown.

B Global superposing of DES-bound ERα-WT and -Y537S LBDs produced an RMSD of 0.5Å. One subunit of each LBD structure is shown (PDB 3ERD and 4ZN7).

C Summary of ERα-Y537S LBD crystal structures obtained. *To be published elsewhere
