# Supplementary material for: Predictive features of ligand‐specific signaling through the estrogen receptor
Source: Mol Syst Biol. 2016 Apr 23;12(4):864. doi: 10.15252/msb.20156701 (PMC4848761; doi:10.15252/msb.20156701)
Supplement: Supplementary file 2 — Expanded View Figures PDF [file MSB-12-864-s002.pdf]

Expanded View Figures

A

Name	Assay type	Reagents	Cell line	Time	Z'
GREB1	mRNA expression by RT-qPCR	TaqMan® gene expression assay Hs00536412_m1	MCF-7	1 day	0.67
E-Luc (native ER)	ERE-driven reporter	3xERE-Luc	Ishikawa	1 day	0.74
L-Luc ERα-WT	ERE-driven reporter	3xERE-Luc, ERα-WT	HepG2	1 day	0.93
L-Luc ERα-ΔAB	ERE-driven reporter	3xERE-Luc, ERα-ΔAB	HepG2	1 day	0.64
L-Luc ERα-ΔF	ERE-driven reporter	3xERE-Luc, ERα-ΔF	HepG2	1 day	0.52
L-Luc ERβ	ERE-driven reporter	3xERE-Luc, ERβ	HepG2	1 day	0.60
NCOA1	M2H interaction-driven reporter	5xUAS-Luc, Gal4-NCOA1 and VP16-ERα	HEK293T	1 day	0.68
NCOA2	M2H interaction-driven reporter	5xUAS-Luc, Gal4-NCOA2 and VP16-ERα	HEK293T	1 day	0.63
NCOA3	M2H interaction-driven reporter	5xUAS-Luc, Gal4-NCOA3 and VP16-ERα	HEK293T	1 day	0.55
Proliferation	Number of cells by luminescence	CellTiter-Glo®	MCF-7	7 days	0.79

B

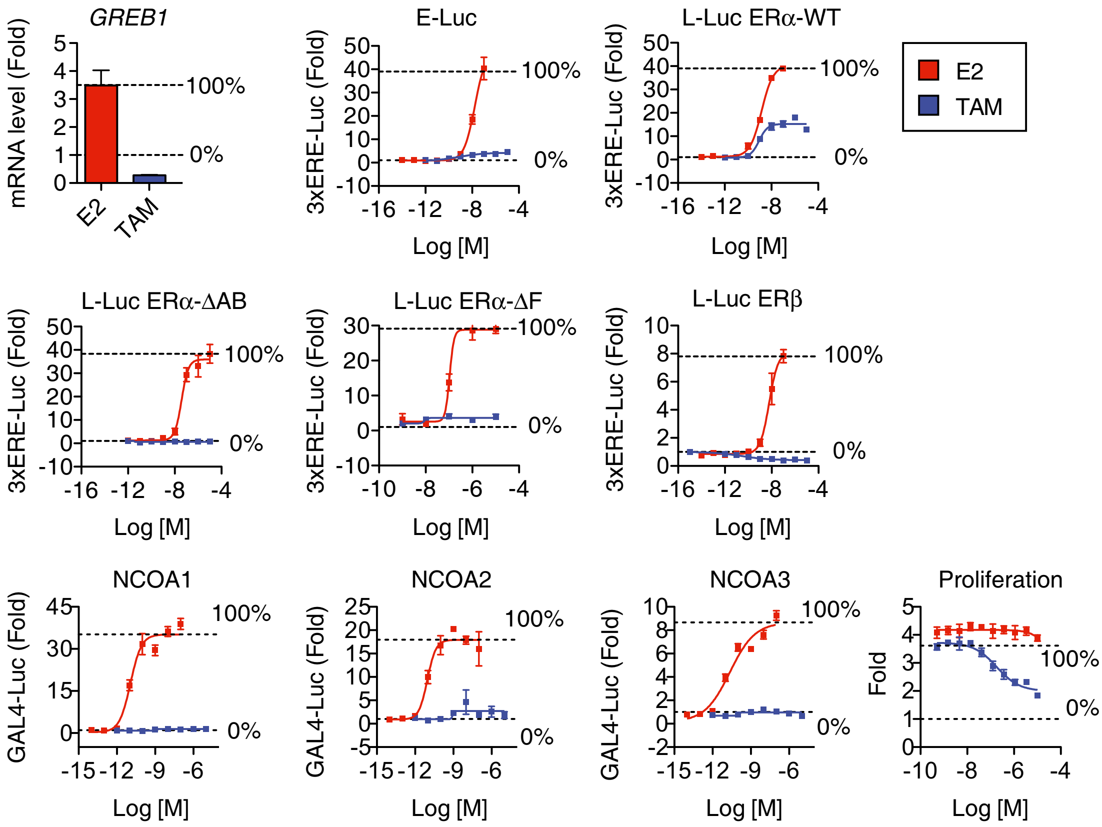

Figure EV1. High-throughput screens for ERα ligand profiling.

A Summary of ligand screening assays used to measure ER-mediated activities. ERE, estrogen-response element; Luc, luciferase reporter gene; M2H, mammalian 2-hybrid; UAS, upstream-activating sequence.

B Controls for screening assays described in panel (A), above. Error bars indicate mean ± SEM, $n = 3$.

© 2016 The Authors

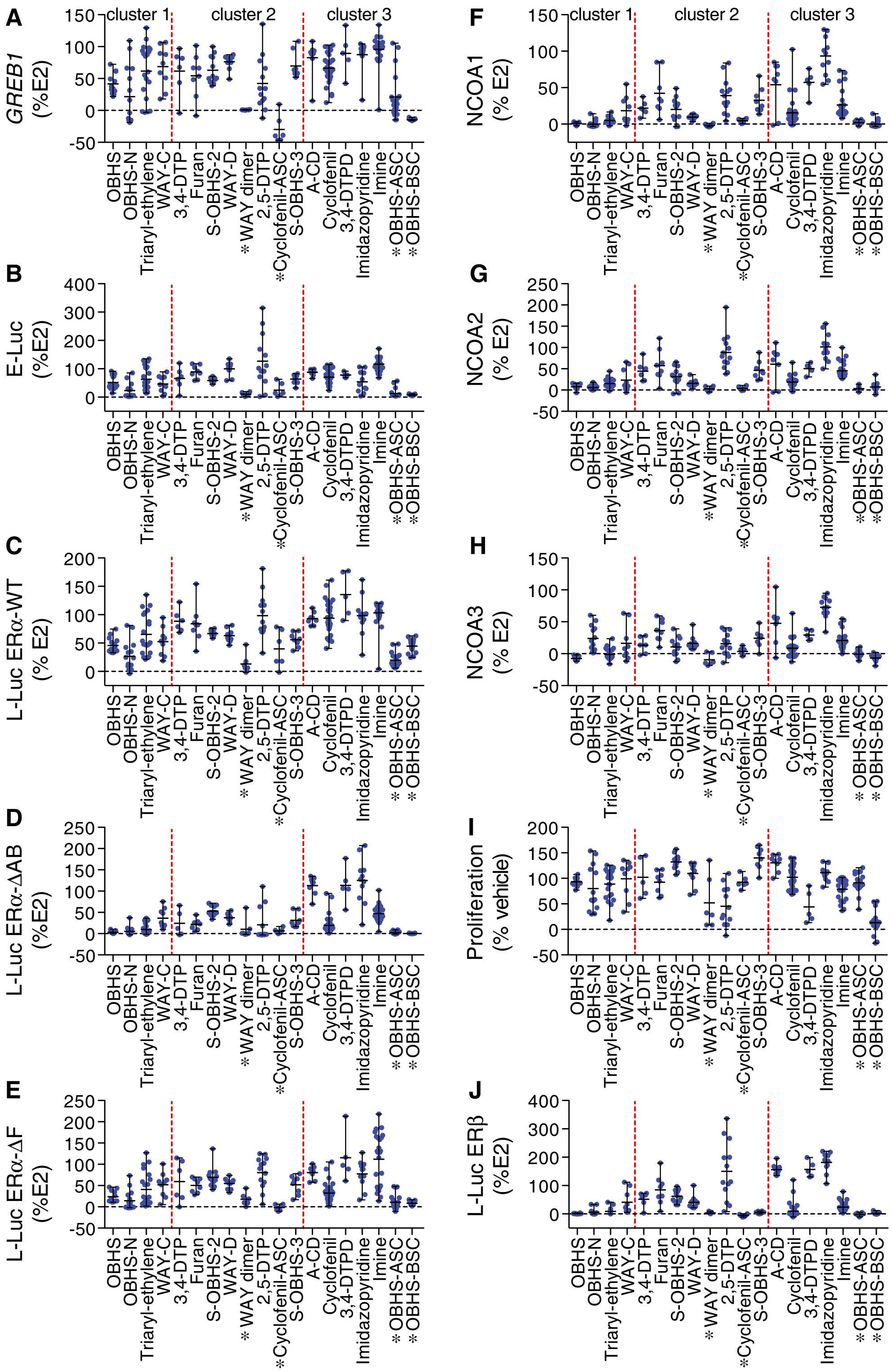

Figure EV2. ERα ligands induced a range of agonist activity profiles.

A–J Screening data from individual ligands are shown, grouped by scaffold. Each data point represents the activity of a distinct compound. Error bars indicate the class average (mean) ± range. *Direct modulator.

Source data are available online for this figure.

© 2016 The Authors

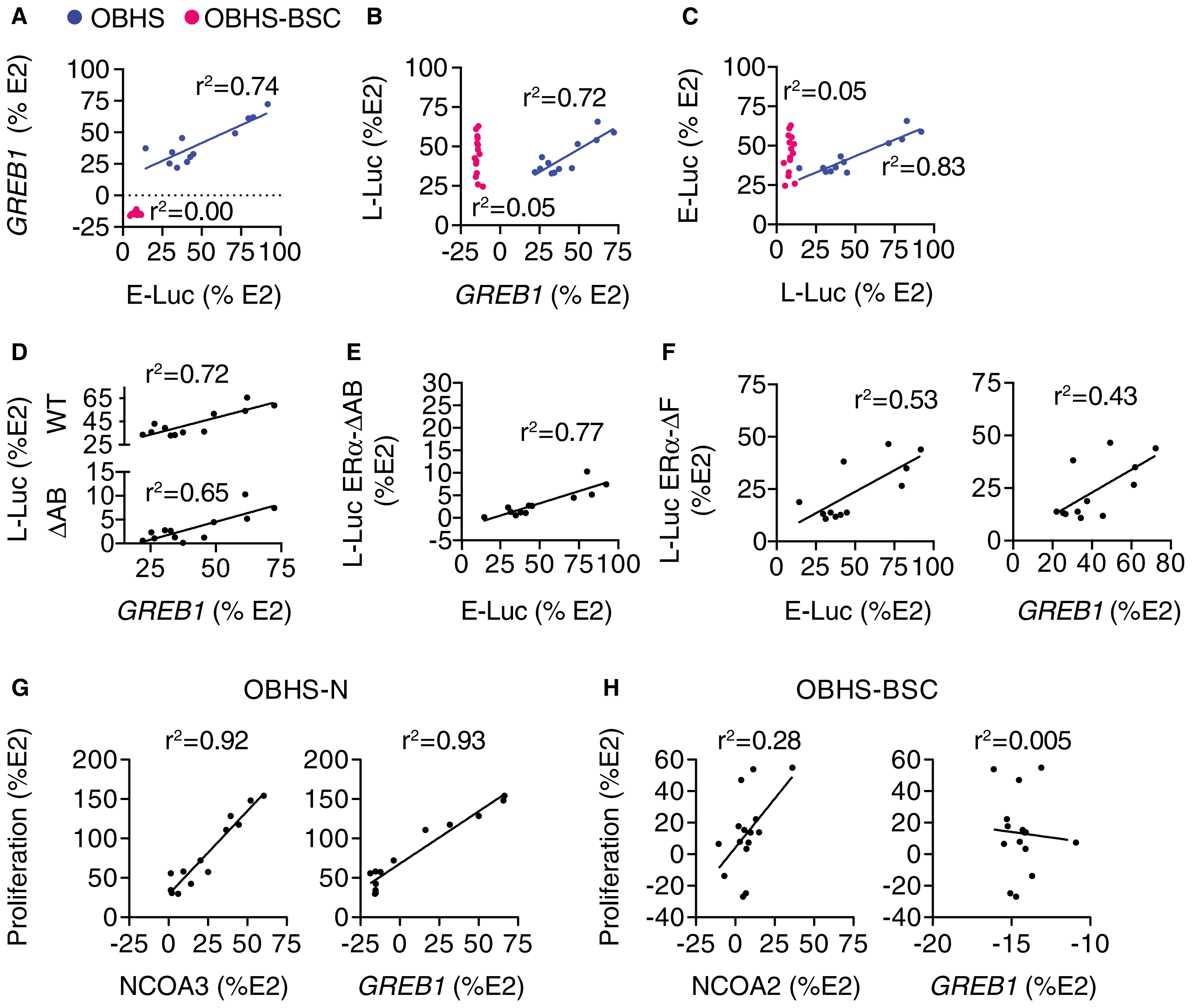

Figure EV3. The side chain of OBHS-BSC analogs induces cell-specific signaling.

A–C Correlation analysis of OBHS versus OBHS-BSC activity across cell types.

D, E Correlation analysis of L-Luc ERα-ΔAB activity versus endogenous ERα activity of OBHS analogs. In panel (D), L-Luc ERα-WT activity from panel (B) is shown for comparison.

F Correlation analysis of L-Luc ERα-ΔF activity versus endogenous ERα activities of OBHS analogs.

G, H Correlation analysis of MCF-7 cell proliferation versus NCOA2/3 recruitment or GREB1 levels observed in response to (G) OBHS-N and (H) OBHS-BSC analogs.

Data information: In each panel, a data point indicates the activity of a distinct compound.
Source data are available online for this figure.

© 2016 The Authors

A

E-Luc vs. L-Luc ERβ

		r^2	p
Cluster 1	OBHS	0.04	5.4E-1
	OBHS-N	0.12	2.5E-1
	Triaryl-ethylene	0.02	5.8E-1
	WAY-C	0.16	2.9E-1
Cluster 2	3,4-DTP	0.03	7.4E-1
	Furan	0.00	9.9E-1
	S-OBHS-2	0.21	1.2E-1
	WAY-D	0.03	6.5E-1
	WAY dimer	0.03	7.3E-1
	2,5-DTP	0.43	1.0E-2*
	Cyclofenil-ASC	0.08	5.8E-1
	S-OBHS-3	0.28	1.8E-1
Cluster 3	A-CD	0.36	1.2E-1
	Cyclofenil	0.22	9.4E-3*
	3,4-DTPD	0.19	4.6E-1
	Imidazopyridine	0.13	2.7E-1
	Imine	0.00	8.1E-1
	OBHS-ASC	0.13	1.2E-1
	OBHS-BSC	0.00	9.1E-1

B

E-Luc vs. L-Luc ERα-WT

	r^2	p
2,5-DTP	0.63	6.0E-4*
Cyclofenil	0.28	2.7E-3*

Figure EV4. ERβ activity is not an independent predictor of E-Luc activity.

A ERβ activity in HepG2 cells rarely correlates with E-Luc activity.

B ERα activity of 2,5-DTP and cyclofenil analogs correlates with E-Luc activity.

Data information: The r^2 and P values for the indicated correlations are shown in both panels. *Significant positive correlation (*F*-test for nonzero slope, *P*-value)

© 2016 The Authors

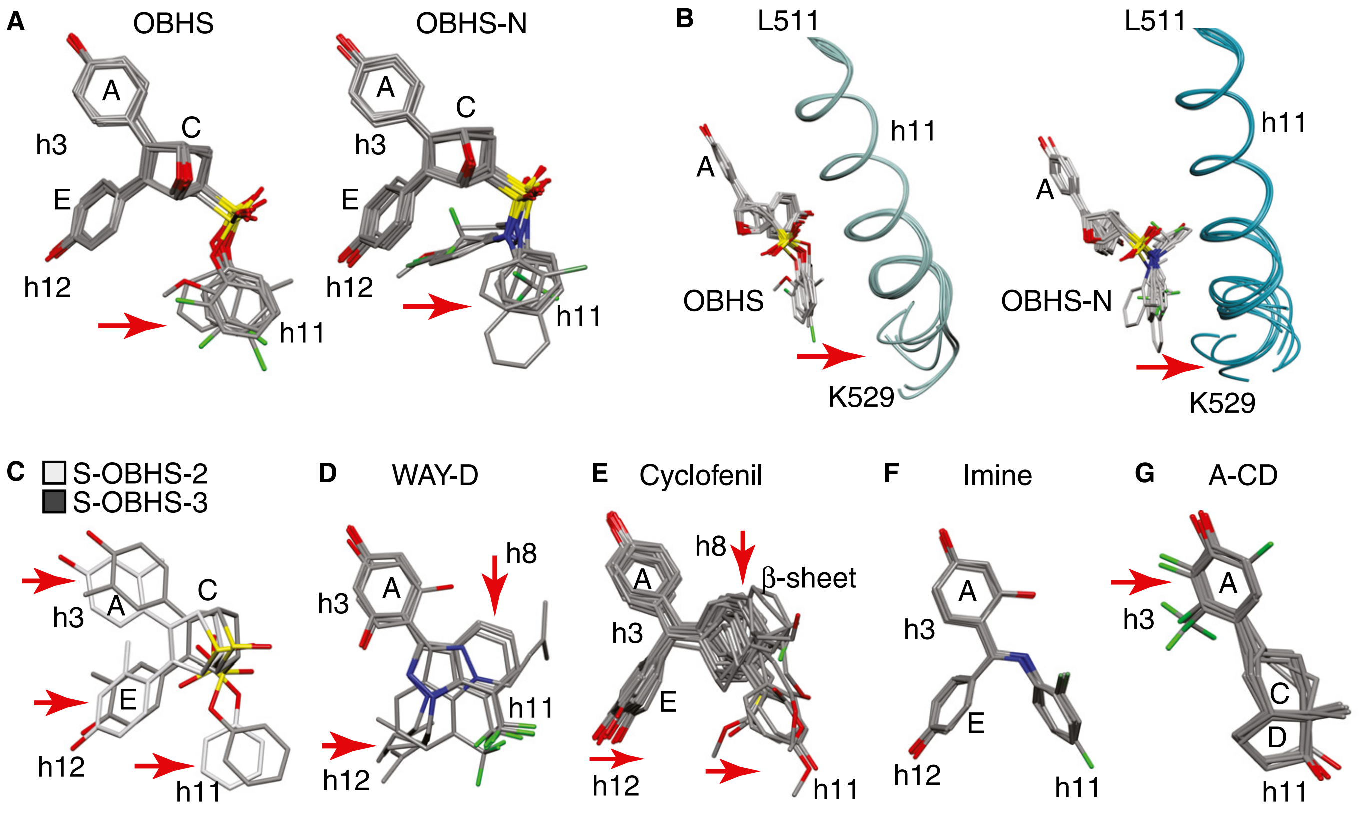

Figure EV5. Structure-class analysis of indirect modulators.

A, B Structure-class analysis of indirect modulators in cluster 1. Crystal structures of the ERα LBD bound to OBHS and OBHS-N analogs were superposed. The bound ligands are shown in panel (A). Arrows indicate chemical variance in the orientation of the different h11-directed ligand side groups. Panel (B) shows the ligand-induced conformational variation at the C-terminal region of h11 (OBHS: PDB 4ZN9, 4ZNH, 4ZNS, 4ZNT, 4ZNU, 4ZNV, and 4ZNW; OBHS-N: PDB 4ZUB, 4ZUC, 4ZWH, 4ZWK, 5BNU, 5BP6, 5BPR, and 5BQ4).

C–G Structure-class analysis of indirect modulators in clusters 2 and 3. Crystal structures of the ERα LBD bound to ligands with cell-specific activities were superposed. The bound ligands are shown, and arrows indicate considerable variation in the orientation of the different h3-, h8-, h11-, or h12-directed ligand side groups.

© 2016 The Authors
